# Activation of axonal Kv7 channels in human peripheral nerve by flupirtine but not placebo - therapeutic potential for peripheral neuropathies: results of a randomised controlled trial

**DOI:** 10.1186/1479-5876-11-34

**Published:** 2013-02-08

**Authors:** Johannes Fleckenstein, Ruth Sittl, Beate Averbeck, Philip M Lang, Dominik Irnich, Richard W Carr

**Affiliations:** 1 Department of Anaesthesiology, Multidisciplinary Pain Center, University of Munich, Pettenkoferstr. 8a, Munich 80336, Germany; 2 Department of Physiology, Ludwig-Maximilians University, Munich, Germany; 3 Department of Anaesthesiology and Intensive Care Medicine, Medical Faculty Mannheim, Heidelberg University, Mannheim, Germany

**Keywords:** Kv7 potassium channel, Flupirtine, Human myelinated axon, A fibre, Randomised controlled trial

## Abstract

**Background:**

Flupirtine is an analgesic with muscle-relaxing properties that activates Kv7 potassium channels. Kv7 channels are expressed along myelinated and unmyelinated peripheral axons where their activation is expected to reduce axonal excitability and potentially contribute to flupirtine’s clinical profile.

**Trial design:**

To investigate the electrical excitability of peripheral myelinated axons following orally administered flupirtine, in-vitro experiments on isolated peripheral nerve segments were combined with a randomised, double-blind, placebo-controlled, phase I clinical trial (RCT).

**Methods:**

Threshold tracking was used to assess the electrical excitability of myelinated axons in isolated segments of human sural nerve in vitro and motoneurones to abductor pollicis brevis (APB) in situ in healthy subjects. In addition, the effect of flupirtine on ectopic action potential generation in myelinated axons was examined using ischemia of the lower arm.

**Results:**

Flupirtine (3-30 μM) shortened the relative refractory period and increased post-conditioned superexcitability in human myelinated axons in vitro. Similarly, in healthy subjects the relative refractory period of motoneurones to APB was reduced 2 hours after oral flupirtine but not following placebo. Whether this effect was due to a direct action of flupirtine on peripheral axons or temperature could not be resolved. Flupirtine (200 mg p.o.) also reduced ectopic axonal activity induced by 10 minutes of lower arm ischemia. In particular, high frequency (ca. 200 Hz) components of EMG were reduced in the post-ischemic period. Finally, visual analogue scale ratings of sensations perceived during the post-ischemic period were reduced following flupirtine (200 mg p.o.).

**Conclusions:**

Clinical doses of flupirtine reduce the excitability of peripheral myelinated axons.

**Trial registration:**

ClinicalTrials registration is NCT01450865.

## Background

Blockade of voltage-dependent sodium channels has been successful in the treatment of some forms of clinical pain, e.g. diabetic polyneuropathy, lumbar radiculopathies, complex regional pain syndrome, neuralgic pain and traumatic peripheral nerve injuries for review: [1]. A group of compounds which might also influence axonal excitability are synthetic activators of slow axonal Kv7 potassium channels, such as flupirtine which has recently been used in the treatment regimens of patients with peripheral neuropathies [2]. The target of such strategies is the K_V_7 (formerly KCNQ/M) family of potassium channels.

Five genes (KCNQ1-5) encode Kv7 subunits, each with 6 trans-membrane spanning domains, a P-loop and a conserved A-domain in the cytoplasmic C-terminal region. Neurones express four Kv7 subunits (Kv7.2-Kv7.5) that co-assemble to form either homo- or hetero-tetrameric voltage-gated channels [3,4] and channel activity can be readily inhibited by agonist action at Gq/11-coupled receptors [5]. In rodents, Kv7.2 is found at nodes of Ranvier in peripheral nerve [6] and in motoneurones in the spinal ventral horn [7], while Kv7.3 is expressed in myelinating Schwann cells [8]. In myelinated axons of smaller calibre, both Kv7.2 and Kv7.3 subunits are found nodally [6] Similarly, Kv7.2, Kv7.3 and Kv7.5 are found along somatic unymelinated axons [9] visceral unmyelinated axons [10] and in baroreceptor nerve terminals [11]. The presence of axonal Kv7 subunits has also been confirmed functionally in peripheral nerve. The synthetic Kv7 channel activator retigabine shifts the activation of myelinated axons in the hyperpolarizing direction [6] and reduces the electrical threshold of unmyelinated axons in human sural nerve [12]. Similarly, flupirtine increases the electrical threshold of myelinated axons in rat sural [13] nerve.

Kv7 channels activate in response to depolarization, beginning at around -60 mV, and deactivate slowly but do not inactivate [4]. The slow time course of voltage activation and lack of inactivation of Kv7 channels suggests that one role of Kv7 in peripheral axons may be to limit the extent of repetitive burst discharges. Consistent with this, the phenotype accompanying dominant-negative mutations in KCNQ2 (Kv7.2) and on occasion KCNQ3 (Kv7.3) is benign familial neonatal convulsions BFNC; [14]. In some patients with BFNC, myokymia symptoms are reported and characterized by spontaneous involuntary muscle fasciculations [7,15-17]. Myokymic symptoms arise from hyperexcitability of motoneurones expressing Kv7.2 [18] and are associated with a point mutation of arginine at position 207 in KCNQ2 that neutralizes the third of six positive charges in the voltage-sensing S4 segment, leading to a slowing and a depolarizing shift of activation [7,17].

Pharmacological activation of Kv7 thus offers an important therapeutic strategy for reducing hyperexcitability. The current study has examined the profile of action of the Kv7 channel activator flupirtine by examining its effects on peripheral myelinated axons at clinically achieved concentrations.

## Materials and methods

### Ethics

Protocol and consent forms were approved by the Committee for Ethics of the University of Munich and ethical approval to study drug effects in healthy volunteers was provided by the German Federal Institute for Drugs and Medical Devices. In accord with the declaration of Helsinki (Seoul 2008) all participants provided their written informed consent prior to participation in the study. ClinicalTrials registration is NCT01450865.

#### In vitro studies

**Procurement and preparation of human sural nerve** Segments of sural and peroneal nerve were obtained from patients previously scheduled for biopsy (n=7; 1 female, 6 male, age range: 48–80). The diagnosis precipitating biopsy was typically polyneuropathy of unknown aetiology. Patients were informed about the procedure by an anaesthesiologist prior to surgery. Upon surgical removal nerve segments were immediately placed in physiological solution containing (in mM): 117 NaCl, 3.6 KCl, 2.5 CaCl_2_, 1.2 MgCl_2_, 1.2 NaH_2_PO_4_, 25 NaHCO_3_, 11 D-glucose (bubbled to pH 7.4 with 95 % O_2_ – 5 % CO_2_).

**Immunohistochemistry** Individual fascicles were isolated from nerve segments and teased apart with fine forceps. After incubating in PBS containing 0.3% collagenase (30 min, room temp.) and washing twice in PBS, preparations were mounted on gelatine-coated slides, dried overnight and fixed in 4% paraformaldehyde for 30 minutes. For immunostaining, preparations were blocked in PBS containing 0.2% Triton 100 and 10% natural goat serum at room temperature for 40 minutes, followed by overnight incubation at 4°C with rabbit anti-KCNQ2-N antibodies diluted 1:200 [19] raised and affinity-purified as previously described [19,20]. KCNQ2 antibodies were directed against residues 13–37 of the conserved intracellular N-terminal region [21,22]. Monoclonal antibodies against peripherin (diluted 1:200) and the alpha subunits of voltage-gated (NaV) sodium channels (panNaV, diluted 1:200) were used. After incubation with the primary antibody, slides were washed three times in PBS and incubated with fluorescein- and rhodamine-conjugated donkey cross-affinity-purified secondary antibodies at room temperature for two hours (all diluted 1:400). Slides were then washed three times with PBS and covered with Aqua-Poly/Mount Coverslipping medium. Digital images were taken with a laser-scanning confocal microscope (Bio-Rad MRC-1024, Bio-Rad Laboratories GmbH, Munich, Germany). No evidence of antibody cross-reactivity was observed in multilabeling experiments with single label and secondary-only controls.

**Threshold tracking** Nerve fascicles were mounted between stimulating and recording electrodes in an organ bath perfused continuously with physiological solution tempered to 32°C. Constant current was used for stimulation (1 ms, A395, WPI, Sarasota, USA) and extracellular signals were amplified (NPI, Tamm, Germany), filtered (30 Hz - 1.3 kHz), sampled (BNC 2120, National Instruments, USA) and stored to disk.

Electrical excitability of myelinated axons was assessed using the TROND^XS4 ^protocol in QTRAC (© Prof. Bostock, Institute of Neurology, London, UK).

### Clinical trial

**Study design** Volunteers were recruited for a randomised, double-blind, placebo-controlled, two-way, cross-over clinical phase I trial (RCT) to investigate the effect of oral flupirtine on the electrical excitability of peripheral myelinated axons. Exclusion criteria were: ongoing medication, prevailing organic disease, previous forearm trauma, primary organ failure, pregnancy or breast-feeding. All female participants were subject to a pregnancy test (Cyclotest, UEBE, Wertheim, Germany) prior to inclusion.

Subjects were assigned randomly to a study arm in accordance with an algorithm developed by the Institute of Medical Information Technology, Biometry and Epidemiology, University of Munich, Germany (IBE), stratifying for gender. Subjects received a set of sealed sequentially numbered pill-boxes each containing either flupirtine or placebo tablets to be taken prior to the recording sessions. Both the subjects and study physicians were blind to the group allocation.

Preliminary results indicated that a sample size of 12 would be needed to detect a decrease in the primary outcome measure (RRP) with 80% power at a significance level of 5%. With provision for drop-outs, 20 subjects were recruited, with an allocation ratio of 1:1.

Participants were evaluated on seven occasions in which motor axon excitability parameters and ischemic EMG signals were recorded (Additional file 1: Figure S1). The first 3 recording sessions, scheduled not less than 48 hours apart, were used to establish baseline values. The 4^th ^recording session comprised 2 sequential recordings of axonal excitability, one immediately prior to pharmacologic intervention and a second two hours after. EMG during ischemia was only recorded after medication. The 5^th ^session was a control recording performed 48 hours after the 4^th^. The 6^th ^recording session was scheduled 7 ± 1 days after the 5th and comprised 2 sequential recordings immediately before and 2 hours after medication. The final (7^th^) recording session was a control recording performed 48 hours after the 6^th^. A pain questionnaire was assessed after the 3^rd^, the 4^th ^and the 6^th ^session.

At the time of examination, subjects lay comfortably in a temperature controlled room.

**Outcome measures** The primary outcome measure was the relative refractory period (RRP) as determined with threshold tracking. All other indices were considered secondary outcome measures.

**Threshold tracking** All experiments were performed by the same examiner. Skin temperature was determined at the wrist at the end of each recording session using a thermocouple (Voltcraft, Hirschau, Germany).

The median nerve of the right hand was used for nerve excitability studies. A gel disc electrode (H92SG, Kendall-Arbo, Neustadt, Germany) positioned at the wrist between palmaris longus and flexor carpi radialis tendons served as the cathode. The anode was placed over the course of the median nerve approximately 2 cm proximal to the cathode. EMG from abductor pollicis brevis (APB) was recorded from one electrode situated over the motor point and a second electrode over the proximal interphalangeal joint.

Constant current pulses (DS5, Digitimer, UK) were used to determine electrical excitability in QTRAC using the TROND^XM4 ^protocol with same measures as for in vitro experiments (see above). The EMG signal was amplified (gain 200-500x), filtered (bandpass 0.3 Hz to 10 kHz) and sampled at 50 kHz (BNC-2120, National Instruments, Austin, USA).

**Ischemia** A cuff around the upper arm inflated to >200mmHg was used to examine flupirtine and placebo effects on ischemic and post-ischemic EMG from APB. Muscle fasciculations are known to appear in some subjects upon recovery from a 10 minute period of ischemia of the lower arm with the intensity and incidence increasing with the period of ischemia [23]. EMG signals were filtered, amplified and sampled at 20 kHz (ADInstruments GmbH, Spechbach, Germany). EMG was recorded continuously over a 21 minute period comprising one minute baseline followed by 10 minutes of ischemia and a further 10 minutes post-ischemia.

Volunteers rated the intensity of sensations before, during and after ischemia on a visual analogue scale (0–100 mm with 0 being no pain and 100 being maximal pain) and completed the Short Form McGill Pain Questionnaire (SF-MPQ) for sensations experienced during the first 5 minutes following release of the pressure cuff, i.e. post-ischemia. The SF-MPQ comprises eleven sensory and four affective pain descriptors that can be ranked in intensity from 0 = *none* to 3 = *severe*[24]. The sum of ranked values provides a sensory (SPRI; 0–33), an affective (APRI; 0–12) and a total pain score (TPRI; 0–45).

### Data analysis

**Axonal excitability** Electrical excitability of myelinated axons determined both in vitro and in vivo were analyzed off-line with the MEM routine in QTRAC. Six parameters were taken directly from the QTRAC analysis, namely the strength-duration time constant, rheobase current, refractoriness at 2 and 2.5 ms, superexcitability at 5 and 7 ms and during the 90-100 ms hyperpolarizing and depolarizing current pulses of the threshold electrotonus. The relative refractory period was determined using custom written software in Igor Pro (WaveMetrics, Lake Oswego, USA) by determining the first zero crossing of an exponential fit to recovery cycle data points less than 5 ms.

**Electromyography** Rectified and integrated EMG was used to quantify continuous signals. EMG power was calculated by discrete fast Fourier transform (FFT) of EMG recorded in the time domain.

### Statistics

Mean and standard deviation are used for population descriptors while mean and standard error of the mean are indicated for comparisons between groups. Normality was tested using Kolmogorov-Smirnov analysis. For parametric datasets Student’s paired t-test was used for pairwise comparisons. Unpaired datasets were compared using one-way ANOVA with post hoc Tukey-Kramer. Non-parametric datasets were analysed using Wilcoxon ranked sign test for paired data, and Kruskal-Wallis ANOVA with post hoc Mann–Whitney-U tests for non-paired data. The level of statistical significance is indicated throughout with * for p < 0.05 and ** for p < 0.01.

### Chemicals and drugs

Triton 100, natural goat serum and the monoclonal antibody against panNaV were purchased from Sigma-Aldrich (Munich, Germany), the monoclonal antibody against peripherin was obtained from Chemicon (EMD Millipore Corporation, Billerica, USA). Anti-KCNQ2/Kv7.2 was kindly provided by M. Schwake (Kiel, Germany). Fluorescein-and rhodamine-conjugated donkey cross-affinity-purified secondary antibodies were purchased from Invitrogen (Paisley, UK). Aqua-Poly/Mount Coverslipping Medium was purchased from Polysciences Europe (Eppelheim, Germany). Flupirtine (in vitro) was purchased from Sigma (Taufkirchen, Germany) and XE991 from Biotrend (Wangen, Switzerland). The final concentration of test substances used in vitro was achieved by diluting stock solutions in the perfusing solution on the day of the experiment.

Blinded oral preparations of flupirtine 100 mg (AWD.pharma GmbH & Co.KG, Radebeul, Germany) and placebo tablets (7 mm capsules (Winthrop Arzneimittel GmbH, Frankfurt, Germany) were performed by the Pharmaceutical Department, University of Munich encapsulating both in gelatine (size 00, white opaque; Shionogl Qualicaps S.A., Alcobendas / Madrid, Spain) containing lactose, ferric oxide and food colouring E172 (all Fagron GmbH, Barsbüttel, Germany).

## Results

### Immunohistochemistry of human A-fibre

**Figure 1:**
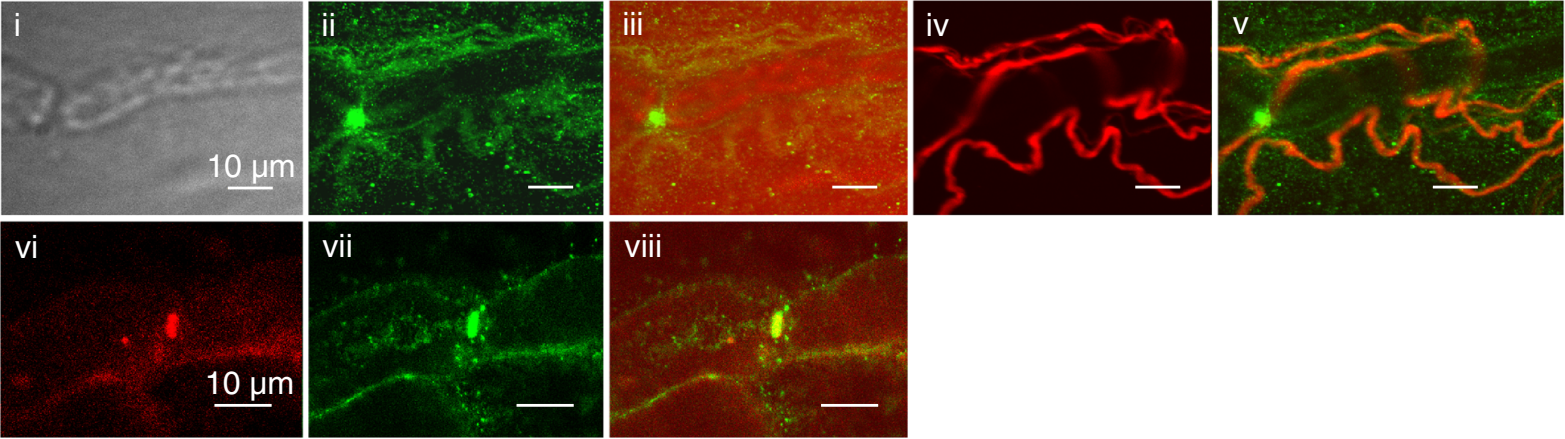
**Immunohistochemistry of Kv7 channels in human sural nerve fascicles *in vitro. ***The presence of Kv7.2 subunits at peripheral nodes of Ranvier as well as in unmyelinated fibres of human sural nerve was verified immunohistochemically. A node of Ranvier evident under bright field illumination (**i**) shows immunoreactivity for Kv7.2 (**ii** and merge **iii**). In addition, unmyelinated fibres labelled with peripherin (**iv**) also show immunoreactivity for Kv7.2 (**ii** and merge **v**). In a separate preparation, Kv7.2 immunoreactivity (**vii**) and immunoreactivity for the non-specific voltage-gated sodium channel marker PanNav (**vi**) colocalise at a node of Ranvier (merge **viii**). Scale bars indicate 10 μm

Using polyclonal antibodies, Kv7.2 subunits could be identified at nodes of Ranvier in teased fibre preparations of human sural nerve (Figure 1). Nodes of Ranvier were identified using DIC images to delineate myelinating Schwann cells and co-labeling with mouse PanNa_V_ (voltage-gated sodium channels, Figure 1 vi-viii). Notably, Kv7.2 was also detected along unmyelinated fibres co-labeled with peripherin (Figure 1 iv, v).

### In vitro assessment of human A-fibre excitability

Recordings were made from twenty-one individual fascicles of human sural nerve. The average compound A-fibre response to supra-maximal electrical stimulation (0.1 ms, 11.03 ± 5.43 mA) was 4.97 ± 1.65 mV and had a latency to half-maximum of 0.82 ± 0.05 ms.

Pooled data for each electrical excitability parameter determined using the TROND^XS4 ^protocol in QTRAC is shown in Table 1. Under control conditions, the strength-duration time constant was 0.11 ± 0.02 μs and the rheobase current 8.53 ± 2.55 μA. ANOVA indicated that neither flupirtine (3-30 μM; strength-duration time constant F (3:43) 2.00, p = 0.13; rheobase F (3:43) 0.22, p = 0.88) nor XE991 (10 μM; strength-duration time constant F (1:26) 1.45, p = 0.24; rheobase F (1:26) 0.27, p = 0.87) had a significant effect on these indices of excitability. Similarly, threshold changes observed during application of depolarizing and hyperpolarizing conditioning currents were also not significantly affected by flupirtine (<30μM, see Table 1).

**Table 1:** **Electrical excitability parameters for myelinated axons in human sural nerve *in vitro***

	**Baseline (n = 21)**			**Flupirtine 3μM (n = 7)**			**Flupirtine 10 μM (n = 8)**			**Flupirtine 30 μM (n = 16)**			**XE 911 10μM (n = 9)**			**ANOVA (df)F, p**
Strength-duration time constant (ms)	0.11	±	0.02	0.19	±	0.03	0.13	±	0.02	0.10	±	0.01	0.16	±	0.03	(3:43)2.00, 0.13
Rheobase (mA)	8.53	±	2.55	6.47	±	2.89	6.50	±	1.76	6.54	±	1.16	9.35	±	1.96	(3:43)0.22, 0.88
Relative Refractory Period RRP (ms)	4.40	±	0.37	3.30	±	0.16	2.83	±	0.17*	2.55	±	0.13**	3.67	±	0.30	(3:48)8.61, **< 0.01**
Refractoriness at 2 ms (%)	76.61	±	21.07	50.58	±	18.13	42.74	±	21.28	45.44	±	21.86	44.03	±	5.21	(3:48)0.57, 0.64
Refractoriness at 2.5 ms (%)	30.43	±	5.63	18.69	±	5.96	10.47	±	7.16*	3.19	±	3.91**	19.46	±	4.29	(3:48)5.39, **< 0.01**
Superexcitability at 5 ms (%)	−6.61	±	1.92	−14.04	±	1.87	−16.20	±	1.18**	−19.00	±	1.180**	−10.83	±	2.30	(3:48)11.56, **< 0.01**
Superexcitability at 7 ms (%)	−11.04	±	1.72	−16.74	±	1.86	−18.01	±	1.27	−20.25	±	1.22**	−14.64	±	2.11	(3:48)7.31, **< 0.01**
Depolarisation TEd (90–100 ms)	34.86	±	1.81	32.48	±	4.11	32.26	±	3.04	32.33	±	2.86	35.96	±	2.33	(3:47)0.27, 0.85
Hyperpolarisation TEh (90–100 ms)	−73.57	±	6.79	−72.99	±	9.23	−68.42	±	5.44	−85.15	±	19.30	−108.79	±	32.65	(3:47)0.27, 0.85

Electrical excitability parameters determined for myelinated axons in human sural nerve in vitro before (Baseline) and during bath application of the Kv7 channel activator flupirtine (3-30 μM) and Kv7 channel blocker XE991 (10 μM). Data were analysed by one-way-ANOVA (rightmost column) and differences for individual groups relative to baseline were determined with post-hoc Tukey-Kramer tests with the levels of significance indicated in each column as * for p < 0.05 and for p < 0.01 respectively. Flupirtine reduced the RRP, the refractoriness at 2.5 ms, the magnitude of post-spike superexcitability at 5 ms and at 7 ms in a concentration-dependent manner but had no effect on the de- and hyperpolarizing threshold electrotonus.

**Figure 2:**
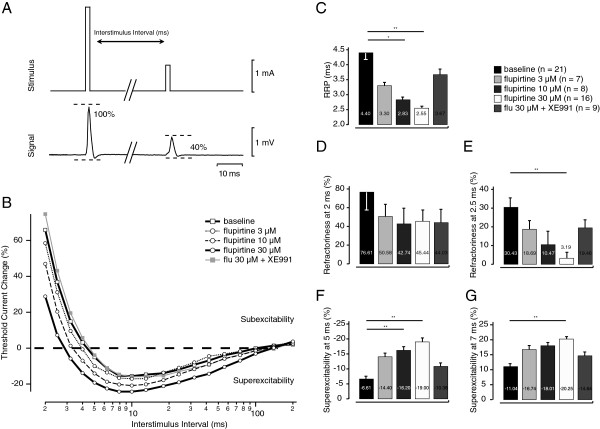
**Effects of Kv7 channel activation and blockade on the recovery cycle of electrical excitability.** Stimulus–response curves were obtained using unconditioned test stimuli of 1 ms duration. These established the maximal CMAP to supramaximal nerve stimulation and the size of the submaximal target CMAP (~ 40% of maximum). The stimulus current necessary to produce the target potential using a 1 ms test stimulus is referred to as the “threshold” for that potential. Electrical excitability, i.e. the current required to maintain a 40% compound A-fibre action potential response, was determined at discrete interstimulus intervals following a supra-maximal stimulus (**A**). The representative example in panel **B** illustrates the concentration-dependent changes in the recovery cycle observed with bath application of flupirtine (3-30 μM) and their reversal following selective Kv7 channel blockade with XE991 (10 μM). Changes in the recovery cycle of A-fibres were quantified with the empirically determined values of refractoriness at 2 ms (**D**) and 2.5 ms (**E**) and excitability at 5 ms (**F**) and 7 ms (**G**). The relative refractory period (**C**) was determined respectively by linear interpolation and first-order exponential fits (see Methods). The RRP (p < 0.01) was reduced in a concentration-dependent manner (**C**). Flupirtine (30 μM) also reduced refractoriness at 2.5 ms (**E**; p < 0.01) but not at 2 ms (**D**). Similarly, flupirtine produced a concentration-dependent increase in the magnitude of post-spike superexcitability at 5 ms (**F**; p < 0.01 for flupirtine 10 μM and 30 μM) and at 7 ms (**G**; p < 0.01; for flupirtine 30 μM)

Flupirtine (10-30 μM) did however reduce the RRP (ANOVA F (3:48) 8.61, p < 0.01) determined in human A-fibres in vitro and this in a concentration-dependent manner (see Figure 2B&C, Table 1; post-hoc Tukey-Kramer p < 0.01for 10 μM and 3 μM). Importantly, these effects could be reversed by bath application of the specific Kv7 channel blocker XE911 (10 μM; Figure 2 B&C and Table 1; ANOVA F (3:36) 7.46, p < 0.01; post-hoc Tukey-Kramer p < 0.05 for flupirtine 30 μM). Flupirtine (30 μM) also reduced refractoriness at 2.5 ms (Figure 2D; ANOVA F (3:48) 5.39, p < 0.01; post-hoc Tukey-Kramer p < 0.01). Similarly, flupirtine produced a concentration-dependent increase in the magnitude of post-spike superexcitability at 5 ms (Figure 2F and Table 1; ANOVA F (3:48) 11.56, p < 0.01; post-hoc Tukey-Kramer p < 0.01 for flupirtine 10 μM and 30 μM) and at 7 ms (Figure 2G; ANOVA F (3:48) 7.31, p < 0.01; post-hoc Tukey-Kramer p < 0.01 for flupirtine 30 μM). Since the RRP is determined from a fit to 3 or more data points, we consider this the most reliable index of changes in excitability.

### In vivo assessment of human A-fibre excitability

Twenty volunteers (10 male, 10 female; age 28.3 ± 4.4 years) were randomly allocated to a study arm and received both placebo and flupirtine (200 mg p.o.) capsules to be taken in a cross-over design. The trial was enroled between October 2009 and August 2010. None of the subjects dropped out, all subjects received the allocated interventions and the follow-up was complete.

Statistical analysis of electrical excitability datasets from the initial three recording sessions revealed no time dependent differences in any of the excitability parameters and further comparisons between this ‘baseline’ dataset and each of the two pre interventional sessions also showed no differences amongst excitability parameters. Consequently, all five baseline datasets were pooled and used as the baseline values for statistical comparisons.

Compound muscle action potential (CMAP) responses were recorded from APB in response to supra-maximal electrical stimulation (4.40 ± 0.22 mA) in 20 volunteers. CMAPs had an amplitude of 8.59 ± 0.54 mV and a latency to half-maximum of 7.40 ± 0.22 ms. The strength-duration time constant was 0.48 ± 0.01 μs and the rheobase current 2.87 ± 0.14 mA. Neither flupirtine (200 mg p.o.) nor placebo affected these measures of excitability (Table 2).

**Table 2:** **Electrical excitability parameters for motoneurones to APB *in vivo *(n = 20)**

	**Baseline**			**Flupirtine 200 mg**			**Placebo**			**Control**			**Paired t-test p, baseline vs. flupirtine**
Strength-duration time constant (ms)	0.48	±	0.01	0.47	±	0.01	0.47	±	0.01	0.47	±	0.01	0.18
Rheobase (mA)	2.87	±	0.14	3.04	±	0.18	2.67	±	0.15	2.82	±	0.14	0.20
Relative Refractory Period (RRP ms)	3.40	±	0.10	3.23	±	0.09	3.45	±	0.12	3.54	±	0.13	**< 0.01**
Refractoriness at 2 ms (%)	165.00	±	13.96	105.83	±	10.12	169.77	±	33.36	138.68	±	9.49	**0.01**
Refractoriness at 2.5 ms (%)	66.27	±	8.86	40.09	±	6.20	60.28	±	8.88	60.21	±	5.84	**0.02**
Superexcitability at 5 ms (%)	−24.57	±	1.49	−24.73	±	1.55	−24.11	±	1.99	−23.28	±	1.54	0.89
Superexcitability at 7 ms (%)	−27.12	±	1.42	−25.69	±	1.67	−27.05	±	1.57	−26.71	±	1.32	0.09
Depolarisation TEd (90–100 ms)	40.04	±	0.78	40.37	±	4.25	40.23	±	1.11	39.74	±	0.96	0.69
Hyperpolarisation TEh (90–100 ms)	−107.61	±	3.01	−106.31	±	2.82	−107.07	±	4.63	−106.30	±	2.92	0.50
Skin Temperature (°C)	31.89	±	0.22	32.20	±	0.29	31.95	±	0.32	31.74	±	0.24	0.13

Electrical excitability parameters determined for motor axons to abductor pollicus brevis in healthy subjects. Values are indicated for recordings made before (Baseline), 2 hours after oral flupirtine (200 mg p.o.) or placebo and 48 hours subsequent to this (post-Control). Skin temperature was determined with a thermocouple placed on the skin at the site of stimulation above the median nerve at the end of each recording session. Data were analysed with Student’s paired t-tests; * and ** indicate levels of significance for p < 0.05 and p < 0.01 respectively. Oral flupirtine reduced the RRP and the refractoriness at 2 ms and 2.5 ms., but had neither effect on the magnitude of superexcitability at both 5 and 7 ms nor the de- and hyperpolarizing threshold electrotonus. There were no changes in peoples skin temperature throughout the experiment.

**Figure 3:**
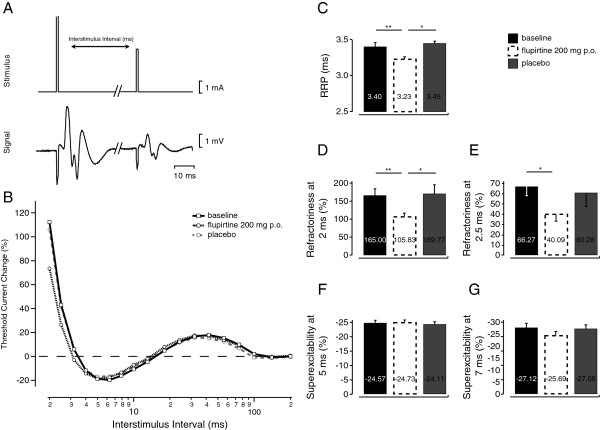
**Effect of oral flupirtine and placebo on the recovery cycle of electrical excitability in motoneurones to abductor pollicis brevis in healthy subjects.** Stimulus–response curves were obtained using unconditioned test stimuli of 1 ms duration. These established the maximal CMAP to supramaximal nerve stimulation and the size of the submaximal target CMAP (~ 40% of maximum). The stimulus current necessary to produce the target potential using a 1 ms test stimulus is referred to as the “threshold” for that potential. Electrical excitability, i.e. the current required to maintain a 40% compound muscle potential response, was determined at discrete interstimulus intervals following a supra-maximal stimulus (**A**). The representative example in panel **B** illustrates the changes in the recovery cycle observed after oral uptake of flupirtine (200 mg) or placebo compared to baseline measures. Changes in the recovery cycle of motor axons were quantified with the empirically determined values of refractoriness at 2 ms (**D**) and 2.5 ms (**E**) and excitability at 5 ms (**F**) and 7 ms (**G**). The relative refractory period (**C**) was determined respectively by linear interpolation and first-order exponential fits (see Methods). RRP in motoaxons to APB (**C**) was reduced 2 hours after oral flupirtine (3.40 ± 0.07 ms; p < 0.01) but not placebo (3.45 ± 0.12 ms, p = 0.53). Oral flupirtine also reduced refractoriness at 2 ms and 2.5 ms (**D**, **E**). In contrast to the effect of flupirtine in vitro, the magnitude of superexcitability at both 5 and 7 ms determined in vivo was not affected by flupirtine (**F**,**G**)

Consistent with observations made in vitro, RRP in motoaxons to APB was reduced 2 hours after oral flupirtine (200 mg p.o.; baseline 3.40 ± 0.07 ms versus flupirtine 3.23 ± 0.09 ms; p < 0.01 paired t-test; see Figure 3B&C and Table 2) but not placebo (3.45 ± 0.12 ms, p = 0.53). Oral flupirtine also reduced refractoriness at 2 ms and 2.5 ms (Figure 3D&E and Table 2). In contrast to the effect of flupirtine in vitro, the magnitude of superexcitability at both 5 and 7 ms determined in vivo was not affected by flupirtine (200 mg p.o; Figure 3F&G). Flupirtine and placebo were also both without effect on de- and hyperpolarizing threshold electrotonus in vivo (Table 2).

### Recordings from APB during ischemia

**Figure 4:**
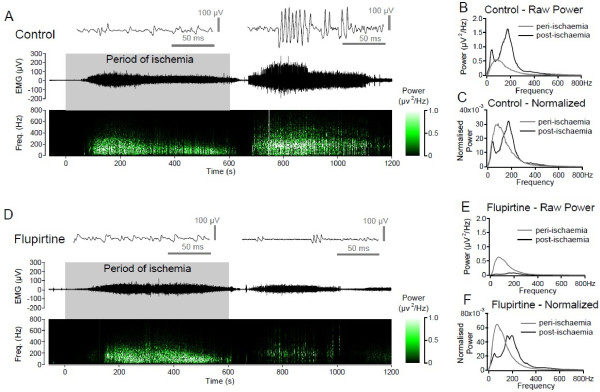
**Effect of oral flupirtine and placebo on postischemic ectopic axonal discharge in motoneurones to abductor pollicis brevis in healthy subjects.** Representative EMG activity recorded from APB in a single subject before during (grey shading) and after a period of ischemia of the lower arm before (**A**, Control) and after a single oral dose of flupirtine (200 mg, **C**). The 10 minute period of ischemia (grey) was produced by inflating a cuff around the upper arm at 200 mmHg for. The upper traces in panels **A**&**D** show raw EMG activity while the lower trace is a running power spectral density (PSD calculated from discrete 2048 point FFTs with 50% overlap and a Hamming window), with power indicated by colour. For comparisons, average PSDs during the 600s peri-ischemic (grey) and post-ischemic (black) periods are shown as absolute power (**B**&**E**) and normalised to total power (**C**&**F**). Under control conditions (A), in the post-ischemic period EMG activity comprises high frequency motor unit discharges (**A**, inset right) and this is reflected in the increased power in the frequency domain around 200 Hz (**B**&**C**). Two hours after a single oral dose (200 mg) of flupirtine (**D**) EMG activity in the peri-ischemic period is little affected, however EMG activity in the post-ischemic period is considerably reduced. The absolute reduction in post-ischemic EMG activity is reflected in the absolute PSD (**E**). Following flupirtine, high frequency motor unit discharges still occur post-ischemically but number of action potentials in these bursts is less (**D**, inset right) and this is reflected in the normalised PSD (**F**) with frequency components around 200 Hz being less prominent than before flupirtine (**C**)

Cuff-induced (200 mmHg) ischemia, induced a substantial increase in surface EMG activity in all 20 subjects (Figure 4A). EMG activity began on average 68.25 ± 8.72 s after cuff inflation and persisted for 288.65 ± 22.93 s. The total rectified integrated EMG during the baseline period of ischemia was 1.08 ± 0.12 V.s and this was reduced to 0.80 ± 0.08 V.s (p < 0.01, paired t-test) 2 hours after flupirtine (200 mg) but not placebo (0.98 ± 0.13 V.s; p = 0.29).

Interestingly, in eleven subjects an increase in surface EMG was also observed in the post-ischemic period (Figure 4A). Post-ischemic EMG activity began on average 125.80 ± 21.11 s after release of the cuff, persisted for 295.20 ± 27.85 s and the total rectified integrated EMG in the 10 minute period following release of the cuff was 0.85 ± 0.10 V.s. Consistent with its effect on EMG activity in the peri-ischemic period, flupirtine also reduced total integrated EMG activity in the post-ischemic period (200 mg p.o.; 0.58 ± 0.05 V.s; p = 0.023 paired t-test, see Figure 4D&E) and this effect was not significant following placebo (0.72 ± 0.09 V.s; p = 0.24).

Under baseline conditions, the surface EMG signal recorded in the post-ischemic period exhibited pronounced high frequency motor unit action potential discharges (MUAP; see insets in Figure 4A&D). The uniformity of MUAP shape and grouping of these discharges suggest that they arose from repetitive discharges in single motor units. The incidence of such high frequency burst discharges was higher in the post-ischemic period than during ischemia. This is evident in the running power spectral density plots shown below the raw EMG traces in Figure 4A&D. Following flupirtine, post-ischemic high frequency repetitive discharges are also evident in surface EMG recordings however the length of each burst was curtailed (Figure 4D, inset). The tendency for flupirtine to curtail high frequency motor unit discharge is shown in the normalised power spectral density plots (PSD; Figure 4C&F) where the relative power in the 150-200Hz band for post-ischemic discharge is reduced following flupirtine (Figure 4F).

### Assessment of sensations during and after ischemia

**Figure 5:**
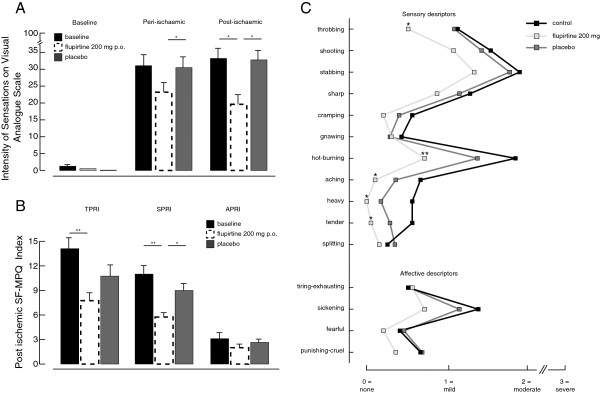
**Subjective rating of sensations perceived during and after ischemia of the arm.** Visual analogue scale ratings (**A**) were determined at rest (Baseline), at the end of a 10 minute period of ischemia (peri-ischemic) and 10 minutes after releasing the cuff (post-ischemic). While at rest there was no significant difference between groups, in the peri- and postischemic phase treatment did have an effect. Especially in the post-ischemic period application of flupirtine (200 mg p.o.) 2 hours prior to the experiment led to a significant decrease in intensity of sensations (-41%, p = 0.03). Sensations perceived in the postischemic period were additionally assessed with the McGill Pain Questionnaire (SF-MPQ; B&C). The total SF-MPQ score (TPRI) comprises both sensory (SPRI) and affective (APRI) components (**B**) and each were determined under control (filled bars) conditions and 2 hours after a single oral dose of flupirtine (open bars) or placebo (grey bars). Flupirtine significantly reduced TPRI (−45%; p < 0.01) and SPRI (-48%; p < 0.01). The SF-MPQ associative verbal descriptors (**C**) were also determined under control conditions (filled) and 2 hours after a single oral dose of flupirtine (200 mg; open) or placebo (grey). Under baseline conditions, the average rating of ‘throbbing’, ‘shooting’, ’stabbing’, ‘sharp’ and ‘hot-burning’ during ischemia was mild-moderate (filled). For the affective descriptors, only ‘sickening’ was rated as moderate. Oral flupirtine (200 mg p.o.) reduced the ratings of ‘hot-burning’ (p < 0.01) and ‘throbbing’(p = 0.04) from moderate to mild. Accordingly to the effect on sensory components, application of flupirtine led to a reduction of several sensory descriptors

Cuff-induced ischemia affects both motor and sensory axons. To assess the sensory component, a visual analogue scale (VAS) was used to rate the intensity of painful sensations during and after ischemia. The VAS comprised a 0 point anchor indicating no pain and an anchor at 100 as the most intense pain imaginable. Averaged across three baseline recordings, pain was rated at 31.1 ± 20.9 mm during ischemia and 33.2 ± 25.4 mm in the postischemic period (Figure 5A). Two hours after oral flupirtine (200 mg) the average ischemic pain rating was 23.2 ± 16.8 mm VAS, (p = 0.12 paired t-test) and 19.6 ± 20.3 mm in the post-ischemic period (-41%, p = 0.03). Oral placebo did not change either the ischemic (30.5 ± 20.6 mm VAS, -1.7%, p = 0.70 paired t-test) nor the post-ischemic pain rating (32.8 ± 27.1 mm VAS, -1.2%, p = 0.69; Figure 5A).

The SF-MPQ was used to assess the intensity and quality of sensations arising in the 10 minute period following release of the cuff. Under baseline conditions, total rating index in the postischemic period was 14.1 ± 1.5 with a sensory and affective component scores of 11.0 ± 1.2 and 3.1 ± 3.0 respectively (Figure 5B). Two hours after flupirtine (200 mg p.o.) the total intensity rating (−45% to 7.8 ± 1.0, p < 0.01, paired t-test) and sensory component scores were reduced (-48%, 5.8 ± 0.7, p < 0.01, paired t-test). In contrast, total intensity ratings were not affected 2 hours after oral placebo (12.0 ± 1.3, p = 0.25, Figure 5B). The SF-MPQ offers descriptors to qualitatively describe sensations. Under baseline conditions, the average rating of ‘throbbing’, ‘shooting’, ’stabbing’, ‘sharp’ and ‘hot-burning’ during ischemia was mild-moderate (Figure 5C). For the affective descriptors, only ‘sickening’ was rated as moderate. Two hours after oral flupirtine (200 mg p.o.) the ratings of ‘hot-burning’ (p < 0.01 Kruskal-Wallis) and ‘throbbing’(p = 0.04) were reduced from moderate to mild.

## Discussion

### Strength and limitations

The primary strength of this study examining drug-induced effects on axonal excitability in vivo and in vitro is the ability to compare and thus judge target engagement and the efficacy of pharmacological compounds on peripheral myelinated axons. In particular, the ability to expose nerve segments to high concentrations provides a basis for the interpretation of subtle changes in excitability detected in people *in situ*, where dose is limited.

Segments of human nerve material were obtained at biopsy and are thus potentially pathological. Previous experiments indicate however that the electrical excitability of nerve fascicles obtained from patients at biopsy is not correlated in a systematic way with the underlying pathology [25,26].

### Generalizability

A common deficit encountered during the clinical and pre-clinical development phases of pharmacological compounds targeting peripheral nerve is the inability to determine directly the axonal effects in people. To address this, standardized measures of axonal excitability were used to examine the effect of flupritine on human peripheral nerve in vitro and in situ. Demonstrable changes in the excitability of myelinated axons were observed *in vitro* (Figure 2) and in motoraxons in situ (Figure 3). This confirmation of target engagement was shown to be therapeutically relevant with flupirtine suppressing ectopic axonal activity evoked by ischemia (Figure 4).

### Interpretation

Kv7.2 subunits were identified nodally in large diameter myelinated axons in human sural nerve (Figure 1) consistent with previous reports using myelinated rat axons [6,8,9]. Kv7.2 expression was also evident along unmyelinated axons [27] where it is also postulated to regulate excitability (6) and suppress axonal discharge [28]. From voltage clamp studies performed on single nodes of Ranvier of myelinated axons from human nerve, it is possible that up to 30% of the slow potassium current may be active at rest [6]. Since, flupirtine shifts the voltage-dependent activation of cell-expressed Kv7.2 in the hyperpolarizing direction [29] it would be expected to hyperpolarize axons by increasing the number of open Kv7 channels at resting membrane potentials around −60 to −70 mV. For the human myelinated axons examined here, flupirtine (3-30μM) produced a concentration-dependent shortening of the relative refractory period and an increase in post-spike superexcitability in vitro, as well as a comparable reduction in the RRP in vivo (2 hours after oral flupirtine).. The increase in post-spike superexcitability seen *in vitro* (Figure 2C) was only apparent for flupirtine concentrations above 10 μM (Figure 2F,G). Previous reports indicate that the peak plasma concentration of flupirtine reaches values in the range of 5 to 6.5 μM, 2 hours after oral flupirtine 200 mg; [30-32]. This concentration is below that at which effects can be detected in vitro and potentially accounts for the lack of effect of flupirtine on the superexcitable phase in motor axons as well as the modest effect of flupirtine on the RRP *in vivo* (Figure 3B,C).

The modest reduction in the value of the relative refractory period in motoraxons to APB may well be secondary to a change in temperature, specifically a warming of peripheral nerve. Propofol was recently reported to shorten the relative refractory period in motoaxons and this effect was attributed to a non-specific increase in skin temperature [33]. Although no global differences in skin temperature were detected between baseline, placebo and flupirtine recording sessions, flupirtine could potentially lead to a warming of peripheral nerve either directly by relaxing vascular smooth muscle [34] or indirectly through suppression of activity in sympathetic neurones [35].

The only other study examining the effects of flupirtine on peripheral axons in people used a 400 mg oral dose and they reported no change in soleus H-reflex latency but a reduction in medium latency flexor reflex responses evoked with 5 pulses at 200 Hz [36]. It is possible that this effect of flupirtine reflects the ability of slow axonal potassium channels to suppress high frequency discharge.

Flupirtine is however also reported to act as an agonist at GABA_A_ receptors in DRG neurones [37]. While an agonist action of flupritine at GABA_A_ receptors may contribute to flupritine’s ability to alter spinal reflex latency, peripheral axons maintain a high intra-axonal chloride concentration and thus activation of GABA_A_ receptors alone cannot account for an increase in post-spike superexcitability or a shortening of the relative refractory period in peripheral myelinated axons.

Interestingly, post-spike recovery cycles recorded from myelinated sural nerve axons in vitro (Figure 1B) appear to lack the late phase of sub-excitability (between ca. 7 and 100 ms) which is otherwise prominent in recovery cycles from median nerve motor axons in vivo (Figure 2B). Recordings in people indicate that late sub-normality is typically smaller in sural nerve than it is in median nerve [38] and that, sural axons thus have less slow nodal potassium channels (Kv7) [38].

Post-ischemic EMG activity arises axonally [39,40] and for a 10 minute period of ischemia affects predominantly myelinated axons [41,42]. In myelinated sensory axons, ectopic activity produces paraesthesias and in motor axons fasciculations are observed [42], with the former being more susceptible due to a more pronounced persistent nodal sodium current [43]. During the period of ischemia, ectopic activity in myelinated axons is typically attributed to a protracted axonal depolarization arising chiefly from a reduction in electrogenic Na^+^/K^+^-ATPase activity and possibly a resultant accumulation of extracellular potassium, particularly in the adaxonal space [44]. Activation of slowly activating, non-inactivating Kv7 channels by flupirtine should adequately counter slow depolarization and indeed flupirtine effectively reduced total EMG activity during ischemia (Figure 4). Post-ischemic EMG activity comprises high frequency components (Figure 4) and microneurographic recordings from myelinated axons in people following ischemia also show prominent high frequency burst discharges ca. 200 Hz; [41,42]. Although high frequency bursts still occurred in the post-ischemic period following flupirtine, there was a reduction in the burst duration, i.e. number of impulses (Figure 4). Flupirtine’s (200 mg p.o) curtailing of action potential burst length in human motoneurones is consistent with previous observations in rat peripheral myelinated axons using retigabine [6] and hippocampal CA1 neurones [45] and suggests that flupirtine may be beneficial in limiting the duration of ectopic bursts in peripheral axons.

Changes in the excitability of peripheral myelinated axons in people (Figures 2 and 3) were comparable with those seen in vitro using human sural nerve segments, suggesting that flupirtine affects Kv7 subunits expressed in peripheral nerve.

## Conclusion and clincial implications

The synthetic Kv7-channel activator flupirtine has been used clinically in Europe for over two decades and its analgesic effects have long been associated with an action on neurones in the CNS. Using a unique combination of in vitro and in vivo methodologies, the clinical profile of flupirtine is shown here to extend to effects on peripheral myelinated axons, reducing their excitability and suppressing aberrant post-ischemic axonal discharge. This highlights the potential benefit of Kv7 channel activators for clinical strategies aimed at reducing hyperexcitability and ectopic discharge in peripheral axons.

## Competing interest

The authors declare that they have no competing interests.

## Authors’ contributions

JF conceived of the study, performed the experiments, analyzed the data and wrote the manuscript. RS conceived of the study, performed the experiments, analyzed the data and wrote the manuscript. BA carried out the immunohistochemical experiments, analyzed the data and and helped to draft the manuscript. PML conceived of the study, and participated in its design and coordination. DI conceived of the study, and participated in its design and coordination. RWC conceived of the study, performed the experiments, analyzed the data and wrote the manuscript. All authors read and approved the final manuscript.

## Supplementary Material

Additional file 1: Figure S1 Study Design. Click here for file
