# Supplementary material for: Activation of axonal Kv7 channels in human peripheral nerve by flupirtine but not placebo - therapeutic potential for peripheral neuropathies: results of a randomised controlled trial
Source: J Transl Med. 2013 Feb 8;11:34. doi: 10.1186/1479-5876-11-34 (PMC3648471; doi:10.1186/1479-5876-11-34)
Supplement: Additional file 1: Figure S1 — Study Design. [file 1479-5876-11-34-S1.docx]

**
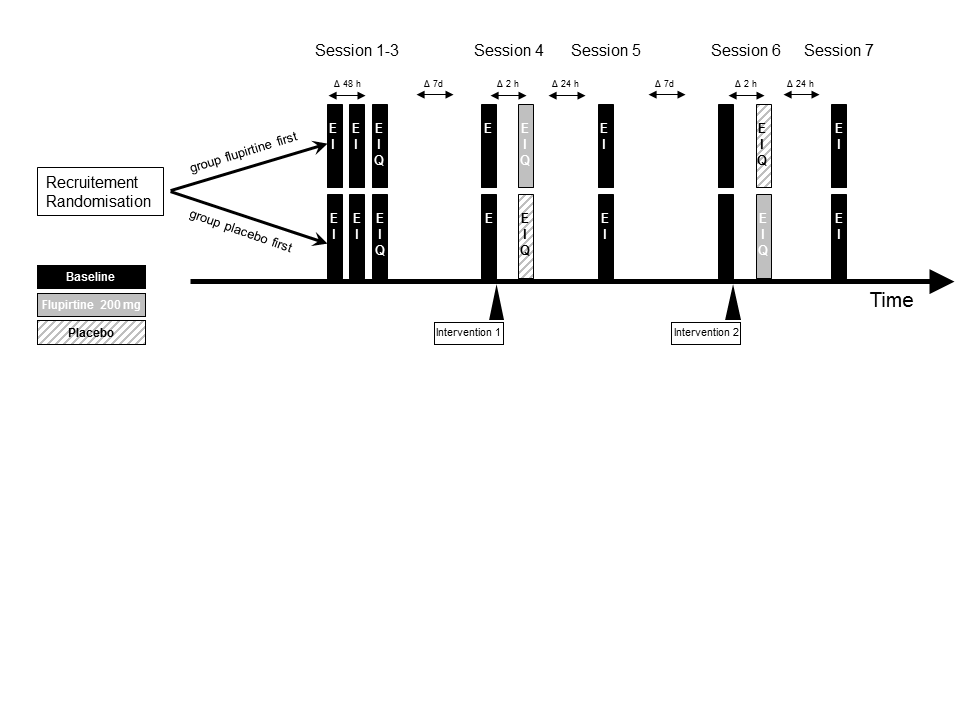
**

**Additional File 1: Study Design.** The Fig. presents the study design as described within the manuscript. Participants were allocated to either flupirtine (grey columns) or placebo (striped columns) administration as first intervention. All participants passed 7 sessions with different recordings: E: threshold tracking, I: ischemic model and Q: Pain Questionnaire.
